# How does hospital organisation influence the use of caesarean sections in low- and middle-income countries? A cross-sectional survey in Argentina, Burkina Faso, Thailand and Vietnam for the QUALI-DEC project

**DOI:** 10.1186/s12884-024-06257-w

**Published:** 2024-01-17

**Authors:** Camille Etcheverry, Ana Pilar Betrán, Myriam de Loenzien, Michael Robson, Charles Kaboré, Pisake Lumbiganon, Guillermo Carroli, Quoc Nhu Hung Mac, Celina Gialdini, Alexandre Dumont, the QUALI-DEC research group, Marion Ravit, Isabella Ramos Mendoza, Newton Opiyo, Meghan Bohren, Charles Kabore, Fadima Yaya Bocoum, Simon Tiendrébéogo, Roger Zerbo, Dittakarn Boriboonhirunsarn, Nampet Jampathong, Kiattisak Kongwattanakul, Ameporn Ratinthorn, Olarik Musigavong, Liana Campodonico, Berenise Carroli, Gabriela Garcia Camacho, Daniel Giordano, Hugo Gamerro, Quoc Nhu Hung Mac, Thao Truong, Tran Minh Thien Ngo, Bui Duc Toan, Huynh Nguyen Khanh Trang, Hoang Thi Diem Tuyet, Claudia Hanson, Helle Molsted-Alvesson, Kristi Sidney Annerstedt, Mariana Romero, Ramon Escuriet, Olga Canet, Karen Zamboni, Laurence Lombard

**Affiliations:** 1 Ceped unit, Université Paris Cité, IRD, Campus Saint-Germain-des-Prés, Inserm, 45 rue des Saints-Pères, Paris, F-75006 France; 2 UNDP/UNFPA/UNICEF/World Bank Special Program of Research, Development and Research Training in Human Reproduction (HRP), Department of Sexual and Reproductive Health and Research, World Health Organization, Geneva, Switzerland; 3 National Maternity Hospital, Dublin, Ireland; 4 Institut de Recherche en Sciences de la Santé, Ouagadougou, Burkina Faso; 5 Department of Obstetrics and Gynaecology, Faculty of Medicine, Khon Kaen University, Khon Kaen, Thailand; 6 Centro Rosarino de Estudios Perinatales, Rosario, Argentina; 7 Pham Ngoc Thach University, Ho Chi Minh City, Vietnam; 8 Facultat de Ciències de la Salut Blanquerna, Universitat Ramon Llull, Barcelona, Spain

**Keywords:** Caesarean section, Hospital organization, Mode of birth, Low- and middle-income countries

## Abstract

**Background:**

Improving the understanding of non-clinical factors that lead to the increasing caesarean section (CS) rates in many low- and middle-income countries is currently necessary to meet the challenge of implementing effective interventions in hospitals to reverse the trend. The objective of this study was to study the influence of organizational factors on the CS use in Argentina, Vietnam, Thailand and Burkina Faso.

**Methods:**

A cross-sectional hospital-based postpartum survey was conducted in 32 hospitals (8 per country). We selected women with no potential medical need for CS among a random sample of women who delivered at each of the participating facilities during the data collection period. We used multilevel multivariable logistic regression to analyse the association between CS use and organizational factors, adjusted on women’s characteristics.

**Results:**

A total of 2,092 low-risk women who had given birth in the participating hospitals were included. The overall CS rate was 24.1%, including 4.9% of pre-labour CS and 19.3% of intra-partum CS. Pre-labour CS was significantly associated with a 24-hour anaesthetist dedicated to the delivery ward (ORa = 3.70 [1.41; 9.72]) and with the possibility to have an individual room during labour and delivery (ORa = 0.28 [0.09; 0.87]). Intra-partum CS was significantly associated with a higher bed occupancy level (ORa = 1.45 [1.09; 1.93]): intrapartum CS rate would increase of 6.3% points if the average number of births per delivery bed per day increased by 10%.

**Conclusion:**

Our results suggest that organisational norms and convenience associated with inadequate use of favourable resources, as well as the lack of privacy favouring women’s preference for CS, and the excessive workload of healthcare providers drive the CS overuse in these hospitals. It is also crucial to enhance human and physical resources in delivery rooms and the organisation of intrapartum care to improve the birth experience and the working environment for those providing care.

**Trial registration:**

The QUALI-DEC trial is registered on the Current Controlled Trials website (https://www.isrctn.com/) under the number ISRCTN67214403.

**Supplementary Information:**

The online version contains supplementary material available at 10.1186/s12884-024-06257-w.

## Background

While the global caesarean section (CS) rate has significantly increased in recent decades (from 7% in 1990 to 21% in 2018) and some regions of Asia or South America present CS rates above 40% and 50% [1, 2], recent trends and projections show that CS rates will continue to rise unequally in the absence of global effective intervention to revert the trend [1]. Between 1990 and 2018, the largest increases in CS rates occurred in Latin America and Asia [1, 2]. Although the increase in CS rates is partly due to an increasing number of institutional deliveries [3], there is a significant increase in the use of CS in health care facilities [3, 4]. Indeed, previous studies on hospital-based CS rates using the Robson classification showed increased rates over time (from 2004 to 2011), and particularly in women in groups 1 to 4 of Robson’s classification which are considered to be low-risk women compared with the other groups [5]. This trend has raised global concern among healthcare providers and governments since there is no evidence of benefit from performing a CS without medical indication [4, 6–8].

Medical indications alone are thus unlikely to explain this phenomenon and the contribution of non-clinical factors in CS use should also be investigated [3, 9, 10]. Factors related to women, healthcare providers or health systems have been proposed to interact and contribute to the increasing use of CS [11]. Women-related factors documented in low- and middle-income countries (LMICs) include socio-economic status or age. Women of higher socio-economic status have a higher probability to give birth by CS compared to poorer women [3, 12–15]. Advanced maternal age also increases the probability of CS [13, 16]. Some antenatal factors such attending antenatal care (ANC) visits in a private facility or a high number of antenatal care (ANC) visits also may influence the mode of birth and the decision for CS [13, 14, 17].

Factors related to healthcare providers have been also identified as influencing the CS use [11, 18–21]. Previous studies have shown that fear of litigation or reputational damage, financial incentives or lack of cooperation and trust between healthcare providers may influence decision-making for CS [18, 22]. However, it appears that physicians often perceive these factors to be associated with the characteristics of the health care systems in which they practice, rather than with their own beliefs or characteristics [18].

Organizational factors associated with CS use have been also investigated [11]. In France, low staffing levels for obstetricians and midwives are associated with higher CS rates [23]. In the United States, hospitals with high profits on CS procedures are more likely to perform CSs [24]. In LMICs, giving birth in the private sector increases the likelihood of having CS in many countries [13, 14, 17, 25–27]. A hospital’s high level of care (ability to provide comprehensive obstetric care) is associated with an increased risk of CS use in Bangladesh [28]. Implementation of clinical practice guidelines with mandatory second opinion or combined with opinion lead, audit and feedback reduced CS rates in some middle-income countries [11]. As organizational factors are potentially suitable for change within the facility, improving understanding of these drivers and how they interact can help envision and develop strategies that support the reduction of unnecessary CS in LMICs.

### The QUALI-DEC project

In response to the growing global importance of non-clinical factors in the rise of CS, a consortium of researchers started the QUALI-DEC project: “Appropriate use of CS through QUALIty DECision-making by women and providers” to implement four non-clinical interventions to reduce unnecessary CS in LMICs [29]: (i) opinion leaders to improve evidence-based clinical practices; (ii) audits and feedback to help healthcare providers identify unnecessary CS; (iii) implementation of companionship during labour and childbirth to support parturient women; (iv) a Decision-Analysis Tool (DAT) to help women make an informed decision on delivery mode. The research project is implemented in 32 health facilities in four countries: Burkina Faso, Argentina, Viet Nam, and Thailand. Despite overall high CS rates in the participating hospitals, there is still some variability in the observed rates. In order to tailor the implementations of these interventions to the local context, it is important to understand the determinants of the mode of birth in the study settings.

With this background, the objective of this study was to assess the influence of organizational factors on the use of CS in a low-risk population before starting the intervention phase of QUALI-DEC project, with the ultimate aim of better tailoring the components of the intervention in each country.

## Materials and methods

### Study design

This study is an ancillary study of the overall QUALI-DEC project, a hybrid efficacy-implementation type III multisite trial registered on the Current Controlled Trials website (number ISRCTN67214403) [29]. The effectiveness of the intervention will be assessed using an interrupted time series analysis and a comparative before-and-after cross-sectional study design. This ancillary study used data from the baseline (pre-intervention) cross-sectional survey. This survey was conducted among a representative sample of postpartum women before discharge from the hospital and will be replicated at the end of the intervention period. The baseline survey took place in all the 32 hospitals participating in the QUALI-DEC project in Burkina Faso, Argentina, Thailand and Vietnam (8 per country). The selection of participating hospitals in each country for the QUALI-DEC project was made purposively for their high CS rates by national or local health authorities in discussion with the research consortium and intended to reflect the diversity of health facilities in each country, regarding their mode of organization.

### Participants and sample size

Women who had delivered a live-born child after 22 weeks’ gestation age (26 weeks in Burkina Faso) were eligible to participate in the baseline cross-sectional survey. Women who had a vital health problem in the post-partum period and those who had given birth to a stillborn child, a neonatal death or a newborn with a malformation were not eligible. Abortions or miscarriages were not considered as deliveries and women who delivered at home or in another health facility (postnatal transfer) were excluded from the survey. The sample size estimate was not calculated specifically for this study but for the effectiveness-implementation research (Quali-Dec). The calculation was based on the expected before-after difference in women’s satisfaction scores, i.e. 470 women per country [29]. The minimum number of women to approach during recruitment was 564 women in each country (71 women per hospital), assuming a 10% non-response rate and 10% ineligible women.

For this study, we selected women who were, a priori, at lower risk of CS: single, full-term pregnancy with cephalic presentation and no previous CS. Women who gave preterm birth (< 37 weeks’ gestation), those with a previous CS, multiple pregnancies or non-cephalic presentations were all excluded. Women with pre-labor emergency CS were also excluded because this type of CS was usually performed for high risk women (e.g. eclampsia, cord prolapse, …).

### Inclusion of participants and data collection

Prior to the cross-sectional survey, a baseline formative research [30] was first conducted in 2020 as part of the QUALI-DEC project. This formative research collected the relevant institutional and organisational data in each hospital to adapt the intervention to the context. This data was collected by each local principal investigator using a data collection form. Data were then extracted by the principal data manager and the final database was controlled by each country principal investigator.

The design of the cross-sectional survey was based on the World Health Organization (WHO) Global Health Survey of Maternal and Perinatal Health [31]. In each country, data collection took place daily in all hospitals until the required number of participants was reached, but still continuing for two full weeks if the sample size was reached earlier. To achieve this goal, we estimated that 5–6 post-partum women had to be interviewed each day. For hospitals with a high volume of activity, a random sampling of women were used to reach 10 selected women per day, among those who had given birth the previous day. We assumed that 4–5 women would refuse to participate or would be ineligible, which would leave us with 5–6 recruitments and interviews per day. The survey period depended on the number of births in each hospital and ranged from 14 days to 46 days between December 2020 and June 2022.

A data collector assigned all randomly selected women an identification number and assessed their eligibility using a screening form. If the eligibility criteria were met, a social scientist approached the post-partum women and offered them to participate during their hospital stay. A consent form was completed when the women agreed to participate and the social scientist interviewed the participants in face-to-face using a tablet for data collection form. The questionnaire was developed based on a literature review and validated by the experts comprising the QUALI-DEC project team. A pilot survey was conducted to test the questionnaire, which was modified where necessary. The information collected was organized in seven modules: women’s characteristics, antenatal care and preference for mode of birth; birth outcomes; women’s knowledge about modes of birth; labour companionship; women’s birth experience and satisfaction; gender dimensions and social equity; wealth characteristics and out-of-pocket expenses. Information on medical history, pregnancy, labour and delivery were simultaneously extracted for all included women by a clinical data collector from medical records using a standard data collection form. Data was entered in duplicate by two local data abstractors into an electronic system specifically designed with validation checks (REDCap®). Final consistency checks were carried out by the principal data manager, with regular support from the country-level data managers.

### Outcomes and explanatory variables

We defined two primary outcomes: (i) pre-labour CS; and (ii) intrapartum CS. The first outcome refers to women who had a planned CS performed before labour as compared to women who attempted a vaginal delivery but ended up with either a vaginal delivery or an emergency intra-partum CS. The second outcome refers to women with a trial of labour but who had an emergency intrapartum CS as compared to women with natural or instrumental vaginal delivery. Furthermore, the rate of instrumental deliveries was very low (1.5% of births) in the 32 hospitals surveyed.

A pre-defined list (Supplementary information Table S1), representative of the main indications for CS in the four participating countries, was used to collect indications for CS, as declared by healthcare providers and recorded in medical records. The various indications for CS were not mutually exclusive (i.e. several indications could be recorded for the same CS).

**Fig. 1:**
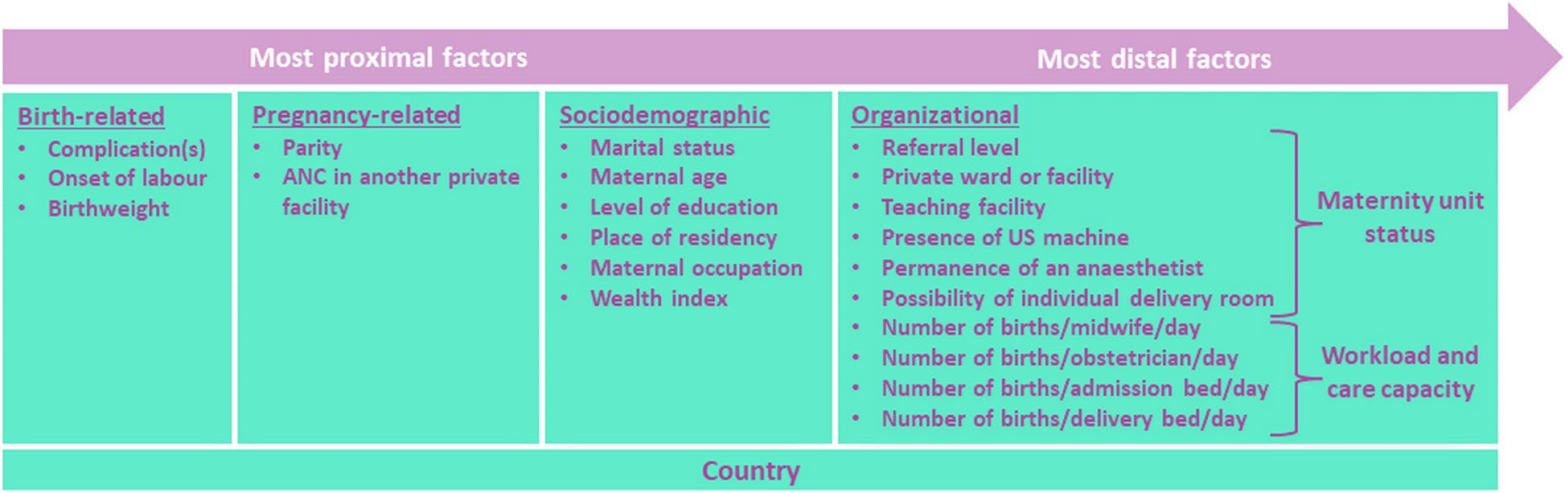
Framework for explanatory variables of CS use in the 32 hospitals (QUALI-DEC project)

We grouped the explanatory variables into women’s characteristics or organizational factors (Fig. 1). The women’s characteristics fall into three categories, starting with the variables that we assumed had a proximal effect on outcomes: (i) birth-related factors; (ii) pregnancy-related factors; and (iii) socio-demographic characteristics. Birth-related factors included the type of labour onset (spontaneous or induced labour), the birthweight (low, normal or high) and pre-existing medical or obstetrical complication that may represent a potential medical need for CS. This last variable is a composite variable assessed at the time of birth, defined as currently having or having had at least one condition from a list of complications extracted from medical records (hypertension and complications; prelabour rupture of membranes; suspected intrauterine growth restriction; diabetes type I/II/gestational; cardiac or renal disease; chronic respiratory conditions; HIV; cholestasis; vaginal bleeding; genital ulcer disease; condyloma accuminata). Pregnancy-related factors included parity, whether or not the woman attended ANC visits in another private facility (outside the hospital where the woman delivered). Sociodemographic factors comprised the place of residency (urban or rural), marital status, maternal age at delivery, maternal level of education, maternal occupation and wealth index. The wealth index was a context-specific composite index, developed through variable selection and component analysis carried out in collaboration with local investigators.

The organizational factors, which we have assumed to have a distal effect on outcomes, included the status of the maternity unit where the woman delivered (academic or not, reference level, totally public or private practice for all/some doctors, functioning ultrasound machine in the delivery ward or not, anaesthetist fully dedicated to the delivery ward or shared with other services, individual delivery room or not), the workload of healthcare providers and the intrapartum care capacity. Workload was modelled as the ratio of the average number of birth per day (annual number of births divided by 365) to the total number of providers (obstetrician or nurse/midwives) working in the maternity unit. The intrapartum capacity was assessed based on the ratio of the average number of births per day (annual number of births divided by 365) to the total number of beds in admission ward or the delivery ward. These organizational factors were selected based of the literature review [13, 14, 16, 18, 23, 32, 33].

Finally, the country was considered as a separate variable, insofar as it captures unmeasured factors, such as those linked to healthcare systems, medical practices or culture of care.

### Data analysis

All analyses were performed using the statistical analysis software Stata/SE® 17. We first described the characteristics of participants using frequencies and percentages for qualitative variables and means and standard deviations (SD) for quantitative variables. Univariate regressions were performed to analyse the crude association between explanatory variables and both primary outcomes.

Multivariable logistic regression models were used to analyse the determinants of having a pre-labour or intrapartum CS. To account for the clustering of outcomes by hospital, a mixed-effects logistic regression models with random intercept were used to present adjusted odds ratios (ORa) and confidence intervals.

A woman’s variable was selected if it was significantly associated to the outcome in bivariate analysis (*p*-value < 0.2). Women’s variables were progressively introduced into the model in the following sequence: (i) birth-related factors; (ii) pregnancy-related factors; (iii) sociodemographic factors. At each step, we selected the variables that provided the most parsimonious model according to the likelihood ratio using a forward stepwise procedure (with a selection of variables having a *p*-value < 0.1). The country of residence was forced into the model. Organizational factors were also forced into the model including maternal characteristics in two steps: one model including all variables related to the status of the maternity unit and another model including variables related to workload and care capacity but excluding variables on maternity status. We tested the interactions between organizational factors and parity because the impact of organization on the use of CS might be different in women with previous experience of birth or not [32]. We also tested this type of interaction regarding women’s country of residence because organization of care was quite different in the four participating countries. We measured the sensitivity of the outcomes to changes in continuous variables (i.e. elasticity). In this way, we used Stata’s *margins* command, which estimates the response margins of the outcome for specified values of covariates. The addition of the *eyex* option allows us to estimate the change in the probability of having a CS caused by a percentage variation (10%) in workload and care capacity.

## Results

A total of 5,840 eligible women gave birth in the 32 hospitals during the data collection period, and 3,127 were randomly selected and provided consent (Supplementary information Figure S1). As per the analysis plan, we further excluded 978 women because of their high risk of undergoing CS and 57 women because they had an emergency pre-labour CS. We then analysed 2,092 women with *a priori* low risk for CS.

**Fig. 2:**
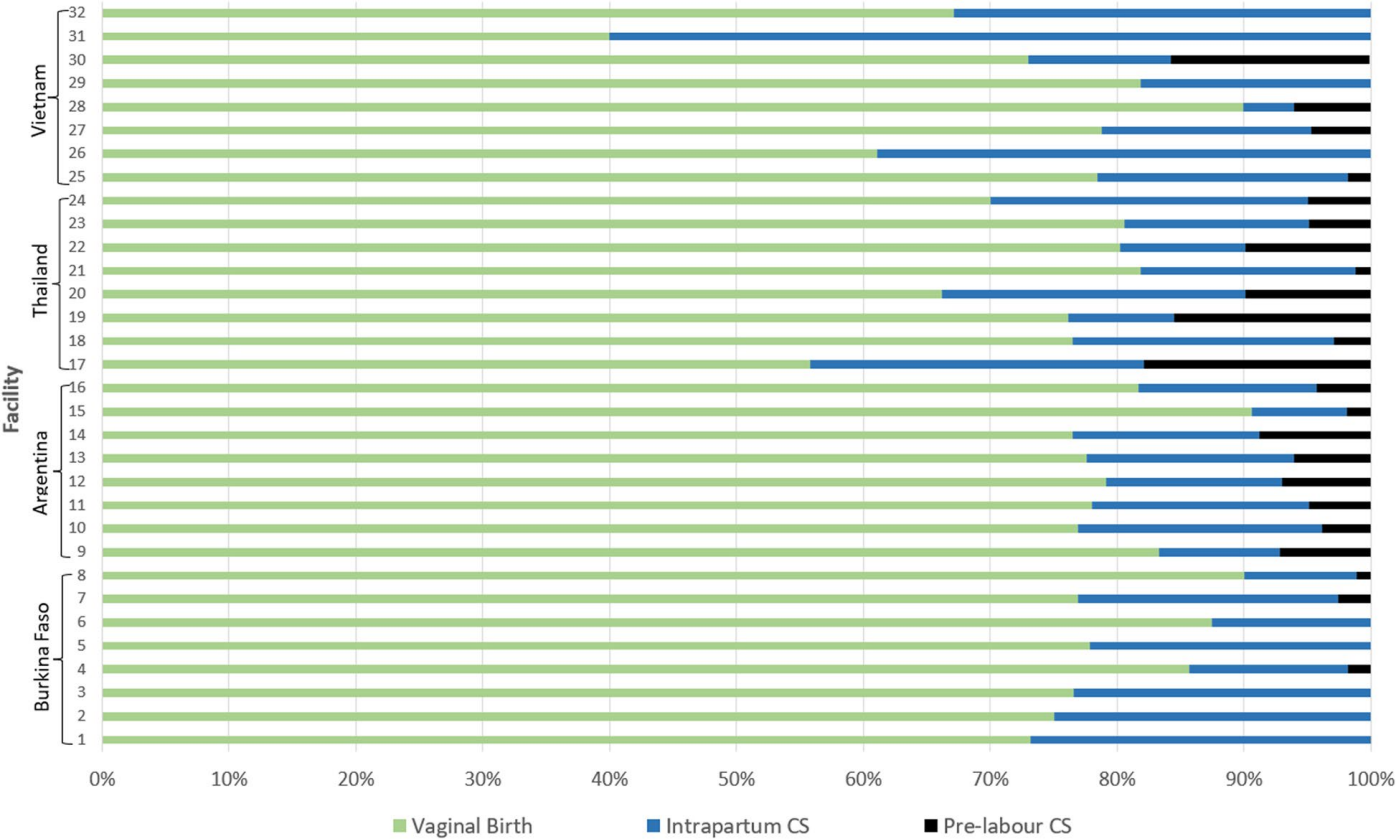
Delivery mode of the 2,092 low-risk women in the 32 participating hospitals. Data collection from December 2020 (Burkina Faso) to June 2022 (Argentina)

Five hundred five participants (24.1%) delivered by CS: 102 women (4.9%) with pre-labour CS and 403 women (19.3%) with intrapartum CS. Pre-labour CS rates by hospital ranged from 0 to 2.6% in Burkina Faso; from 1.9 to 8.8% in Argentina; from 1.2 to 17.9% in Thailand; and from 0 to 15.7% in Vietnam. Intrapartum CS rates by hospital ranged from 8.9 to 26.8% in Burkina Faso; from 7.5 to 19.2% in Argentina; from 8.4 to 26.3% in Thailand; and from 4 to 60% in Vietnam (Fig. 2).

**Fig. 3:**
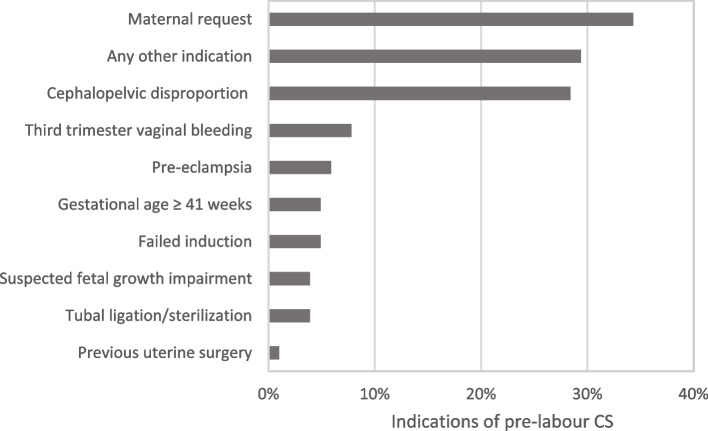
Indications of pre-labour CS in low-risk women (*n* = 102, QUALI-DEC)

**Fig. 4:**
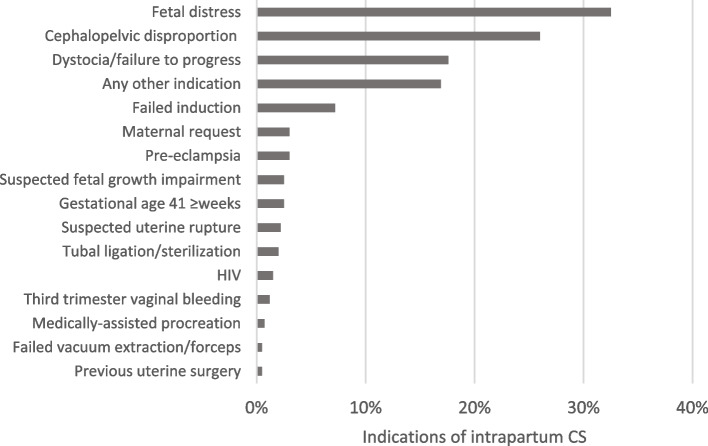
Indications of intrapartum CS in low-risk women (*n* = 403, QUALI-DEC)

In more than 30% of pre-labour CSs, the maternal request was one of the indications (Fig. 3). The most common indications for intrapartum CS were fetal distress (30%), cephalopelvic disproportion (26%) and dystocia/failure to progress (17.6%) (Fig. 4).

Women’s characteristics and organizational factors are presented in Tables 1 and 2, respectively. Most hospitals were teaching facilities with functioning ultrasound devices in the delivery ward. In contrast, few of them had a 24-hour anaesthetist dedicated to the maternity unit and offered women the possibility of giving birth in an individual delivery room.

**Table 1:** Characteristics of low-risk women (QUALI-DEC)

Women’s characteristics	Total (*N* = 2092)
**Complication at delivery** ^a^ **, n (%)**	
No (ref)	1608 (76.9)
Yes	484 (23.1)
**Onset of labour, n (%)**	
Spontaneous (ref)	1768 (84.5)
Induced	222 (10.6)
No labour (pre-labour CS)	102 (4.9)
**Birth weight, n (%)**	
Low (< 2500 g)	81 (3.9)
Normal (2500-4000 g) (ref)	1917 (91.6)
Macrosomia (≥ 4000 g)	94 (4.5)
**Parity, n (%)**	
Nulliparous (ref)	958 (45.8)
Multiparous	1133 (54.2)
**Attending ANC in another private facility, n (%)**	
No (ref)	1254 (59.9)
Yes	838 (40.1)
**Country, n (%)**	
Burkina Faso (ref)	407 (19.5)
Argentina	440 (21.0)
Thailand	572 (27.3)
Vietnam	673 (32.2)
**Marital status, n (%)**	
Married/Living with a partner (ref)	1960 (93.7)
Separated/Single/Widow	132 (6.3)
**Maternal age, n (%)**	
< 25 years (ref)	702 (33.6)
25–35 years	1085 (51.9)
≥ 35 years	305 (14.6)
**Level of education, n (%)**	
Secondary and less (ref)	1489 (71.2)
University	603 (28.8)
**Place of residency, n (%)**	
Rural (ref)	567 (27.2)
Urban	1514 (72.8)
**Maternal occupation, n (%)**	
Unemployed/Housewife (ref)	786 (37.6)
Employed formal sector	574 (27.4)
Informal sector	731 (35.0)
**Wealth index, n (%)**	
Poorest (ref)	451 (21.6)
Poorer	467 (22.3)
Middle	521 (24.9)
Richer	314 (15.0)
Richest	339 (16.2)

^a^At least one of the following complications: hypertension and related complications (*n* = 127); prelabour rupture of membranes (*n* = 223); suspected fetal growth impairment (*n* = 22); diabetes type I/II/gestational (*n* = 133); cardiac or renal disease (*n* = 9); chronic respiratory conditions (*n* = 4); HIV (*n* = 9); cholestasis (*n* = 4); vaginal bleeding (*n* = 1); condyloma accuminata (*n* = 5)

**Table 2:** Institutional variables of the hospitals where the study women delivered (QUALI-DEC)

Institutional variables^a^	Argentine (*N* = 8)	Burkina Faso (*N* = 8)	Thailand (*N* = 8)	Vietnam (*N* = 8)	Total of women (*N* = 2092)
**Referral level, n (%)**					
Primary – Secondary (ref)	2 (25.0)	6 (75.0)	1 (12.5)	7 (87.5)	1119 (53.5)
Tertiary	6 (75.0)	2 (25.0)	7 (87.5)	1 (12.5)	973 (46.5)
**Any private practice in the maternity unit, n (%)**					
No (ref)	7 (87.5)	2 (25.0)	2 (25.0)	2 (25.0)	971 (46.4)
Yes (private ward or facility)	1 (12.5)	6 (75.0)	6 (75.0)	6 (75.0)	1121 (53.6)
**Teaching facility, n (%)**					
No (ref)	0 (0.0)	5 (62.5)	0 (0.0)	2 (25.0)	468 (22.4)
Yes	8 (100.0)	3 (37.5)	8 (100.0)	6 (75.0)	1624 (77.6)
**Presence of a functioning US machine in delivery ward, n (%)**					
No (ref)	4 (50.0)	4 (50.0)	0 (100.0)	1 (12.5)	513 (24.5)
Yes	4 (50.0)	4 (50.0)	8 (100.0)	7 (87.5)	1579 (75.5)
**Permanence of an anaesthetist, n (%)**					
No (ref)	7 (87.5)	7 (87.5)	2 (25.0)	4 (50.0)	1151 (55.0)
Yes	1 (12.5)	1 (12.5)	6 (75.0)	4 (50.0)	941 (45.0)
**Any individual delivery room, n (%)**					
No (ref)	6 (75.0)	8 (100.0)	7 (87.5)	6 (75.0)	1564 (74.8)
Yes	2 (25.0)	0 (0.0)	1 (12.5)	2 (25.0)	528 (25.2)
**Number of births per one midwife/nurse per day, median (Q1-Q3)**	0.7 (0.5–0.8)	1.4 (1.1–1.7)	0.9 (0.8–1.0)	1.2 (0.5–1.6)	1.0 (0.8–1.4)
**Number of births per one obstetrician per day, median (Q1-Q3)**	0.7 (0.6–0.8)	5.1 (4.0–6.9)	4.5 (3.5–6.6)	2.9 (2.1–4.0)	3.7 (2.0–4.9)
**Number of births per admission bed per day, median (Q1-Q3)**	0.8 (0.7–1.3)	2.3 (1.7–3.1)	0.8 (0.7–0.9)	1.8 (0.9–2.7)	1.1 (0.8–2.1)
**Number of births per delivery bed per day, median (Q1-Q3)**	1.0 (0.9–1.8)	2.3 (1.7–3.1)	2.6 (1.4–3.1)	2.1 (1.8–2.9)	2.1 (1.4–3.0)

^a^Characteristics of the hospital where the woman delivered

*US *Ultrasound

Most women’s and institutional characteristics were significantly associated to both outcomes in bivariate analysis with *p*-value less than 0.2 (Table 3). Regarding women’s characteristics, only marital status was excluded from the multivariable models for pre-labour CS. Marital status and maternal age were excluded from the models for intrapartum CS.

**Table 3:** Women’s characteristics and organizational factors associated with pre-labour and intrapartum caesarean delivery: bivariate analysis (QUALI-DEC)

		Pre-labour caesarean delivery *N* = 102/2092				Intrapartum caesarean delivery *N* = 403/1990			
		OR (95% CI)^a^		*p*-value		OR (95% CI)^a^		*p*-value	
**Complication at delivery**		2.2 (1.5–3.4)		< 0.001		1.7 (1.3–2.2)		< 0.001	
**Induction of labour**		-		-		1.6 (1.1–2.2)		< 0.01	
**Birth weight**				< 0.001				0.04	
Low (< 2500 g)		1.1 (0.4–3.2)		0.81		0.9 (0.5–1.6)		0.72	
Normal (2500-4000 g)		1				1			
Macrosomia (≥ 4000 g)		3.8 (2.1–7.0)		< 0.001		1.8 (1.1–3.0)		0.01	
**Nulliparity**		1.7 (1.2–2.6)		< 0.01		3.2 (2.5–4.0)		< 0.001	
**Attending ANC in another private facility**		2.0 (1.3–2.9)		0.001		1.3 (1.1–1.7)		< 0.01	
**Country**				< 0.001				< 0.001	
Burkina Faso		1				1			
Argentina		7.9 (2.3–26.8)		0.001		0.8 (0.5–1.1)		0.17	
Thailand		14.6 (4.5–47.0)		< 0.001		1.1 (0.8–1.5)		0.55	
Vietnam		5.8 (1.8–19.4)		< 0.01		1.5 (1.1–2.0)		< 0.01	
**Marital status**									
Separated/Single/Widow		0.9 (0.4–2.1)		0.85		1.0 (0.6–1.5)		0.91	
**Maternal age**				< 0.001				0.58	
< 25 years		1				1			
25–35 years		1.6 (0.9–2.6)		0.07		1.1 (0.8–1.4)		0.55	
≥ 35 years		3.0 (1.7–5.4)		< 0.001		0.9 (0.6–1.3)		0.59	
**University level of education**		3.1 (2.1–4.6)		< 0.001		1.6 (1.3–2.0)		< 0.001	
**Urban residency**		1.5 (0.9–2.4)		0.12		0.8 (0.6–1.0)		0.04	
**Maternal occupation**				0.01				< 0.001	
Unemployed/housewife		1				1			
Employed formal sector		1.5 (0.9–2.4)		0.08		1.5 (1.2–2.0)		< 0.01	
Informal sector		0.7 (0.4–1.2)		0.21		0.8 (0.6–1.1)		0.11	
**Wealth index**				0.08				0.06	
Poorest		1				1			
Poorer		1.5 (0.7–3.1)		0.26		1.0 (0.7–1.5)		0.77	
Middle		1.8 (0.9–3.6)		0.08		1.5 (1.1–2.1)		0.01	
Richer		1.9 (0.9–4.0)		0.08		1.3 (0.9–1.8)		0.21	
Richest		2.7 (1.3–5.3)		< 0.01		1.0 (0.7–1.5)		0.90	
**Status of the maternity unit**									
Tertiary level of care		1.7 (1.1–2.5)		0.01		1.0 (0.8–1.2)		0.99	
Private practice in the maternity unit		2.1 (1.3–3.2)		0.001		1.5 (1.2–1.9)		< 0.001	
Teaching facility		0.6 (0.3–1.0)		0.06		0.9 (0.7–1.2)		0.57	
Functioning US machine in delivery ward		1.9 (1.1–3.4)		0.02		1.4 (1.1–1.9)		< 0.01	
Permanence of an anaesthetist		3.4 (2.2–5.3)		< 0.001		1.2 (0.9–1.5)		0.13	
Any individual delivery room		0.7 (0.4–1.2)		0.18		1.1 (0.9–1.4)		0.36	
**Workload and care capacity**									
Number of births per one midwife/nurse per day		0.9 (0.6–1.3)		0.43		0.7 (0.6–0.9)		< 0.01	
Number of births per one obstetrician per day		1.0 (0.9–1.1)		0.42		1.0 (0.9–1.0)		0.20	
Number of births per admission bed per day		0.7 (0.6–0.9)		0.02		0.9 (0.8–1.0)		0.11	
Number of births per delivery bed per day		0.9 (0.7–1.2)		0.61		1.2 (1.1–1.4)		0.001	

^a^Crude Odds Ratio (95% confidence interval) using univariate logistic regression; US: Ultrasound

Taking into account women’s characteristics, regression model 2 shows that pre-labour CS was significantly associated with a 24-hour anaesthetist dedicated to the delivery ward (ORa = 3.70 [1.41; 9.72], *p* < 0.01) and with the possibility to have an individual room during labour and delivery (ORa = 0.28 [0.09; 0.87], *p* = 0.03) (Table 4). Workload and care capacity indicators (model 3) were not significantly associated with the use of pre-labour CS.

**Table 4:** Multilevel logistic regression models^a^ for the association of pre-labour caesarean delivery with women’s characteristics and organizational factors (*N* = 2,092, QUALI-DEC)

Variables	Adjusted odds rations (95% Confidence interval)					
	Model 1^b^	*p*-value	Model 2^b^	*p*-value	Model 3^b^	*p*-value
**Country**		< 0.01		< 0.01		< 0.01
Burkina Faso	1		1		1	
Argentina	6.57 (1.41–30.7)	0.02	22.3 (3.16–157.2)	< 0.01	12.3 (1.47–103.9)	0.02
Thailand	8.40 (1.84–38.2)	< 0.01	7.15 (1.05–48.7)	0.04	27.1 (4.20–175.4)	0.001
Vietnam	2.11 (0.44–10.2)	0.35	2.42 (0.46–12.8)	0.30	2.91 (0.53–16.1)	0.22
**Complication at delivery**	1.97 (1.23–3.15)	< 0.01	1.88 (1.18–3.0)	< 0.01	2.01 (1.26–3.21)	< 0.01
**Birth weight**		< 0.001		< 0.001		< 0.001
Low (< 2500 g)	0.65 (0.20–2.07)	0.47	0.67 (0.21–2.13)	0.50	0.65 (0.20–2.07)	0.47
Normal (2500-4000 g)	1		1		1	
Macrosomia (≥ 4000 g)	7.08 (3.42–14.6)	< 0.001	7.11 (3.43–14.7)	< 0.001	6.85 (3.32–14.1)	< 0.001
**Nulliparity**	2.35 (1.40–3.94)	0.001	2.30 (1.38–3.85)	0.001	2.37 (1.42–3.97)	0.001
**Attending ANC in another private facility**	2.28 (1.35–3.86)	< 0.01	2.32 (1.37–3.92)	< 0.01	2.32 (1.37–3.92)	< 0.01
**Maternal age**		< 0.01		< 0.01		0.01
< 25 years	1		1		1	
25–35 years	1.38 (0.76–2.49)	0.28	1.37 (0.76–2.47)	0.29	1.39 (0.78–2.50)	0.27
≥ 35 years	2.90 (1.40–6.0)	< 0.01	2.92 (1.42–6.01)	< 0.01	2.87 (1.39–5.91)	< 0.01
**University level of education**	1.79 (1.08–2.95)	0.02	1.81 (1.10–2.97)	0.02	1.80 (1.09–2.96)	0.02
**Status of the maternity unit**						
Tertiary level of care	-	-	0.99 (0.36–2.71)	0.99	-	-
Private practice in the maternity unit	-	-	0.46 (0.16–1.31)	0.15	-	-
Teaching facility	-	-	1.83 (0.47–7.12)	0.38	-	-
Functioning US machine in delivery ward	-	-	1.81 (0.55–5.93)	0.33	-	-
Permanence of an anaesthetist	-	-	3.70 (1.41–9.72)	< 0.01	-	-
Any individual delivery room	-	-	0.28 (0.09–0.87)	0.03	-	-
**Workload and care capacity**						
Number of births per one midwife/nurse per day	-	-	-	-	1.86 (0.66–5.23)	0.24
Number of births per one obstetrician per day	-	-	-	-	0.96 (0.70–1.31)	0.78
Number of births per admission bed per day	-	-	-	-	1.68 (0.81–3.51)	0.16
Number of births per delivery bed per day	-	-	-	-	0.73 (0.44–1.22)	0.23
**Intraclass correlation**	-	-	0.07 (0.02–0.28)		0.13 (0.05–0.30)	

^a^Mixed-effects logistic regression models with random intercept

^b^Model 1: women’s characteristics; model 2: women’s characteristics and maternity unit status; model 3: women’s characteristics and indicators of workload and care capacity

Model 6 shows that intra-partum CS was significantly associated with the average number of births per delivery bed per day: ORa = 1.45 [1.09; 1.93], *p* < 0.01 (Table 5). The other organizational factors had no effect on intra-partum CS rates.

**Table 5:** Multilevel logistic regression models^a^ for the association of intrapartum caesarean delivery with women’s characteristics and organizational factors (*N* = 1,990, QUALI-DEC)

Variables	Adjusted odds rations (95% Confidence interval)					
	Model 4^b^	*p*-value	Model 5^b^	*p*-value	Model 6^b^	*p*-value
**Country**		0.54		0.59		0.17
Burkina Faso	1		1		1	
Argentina	0.63 (0.32–1.23)	0.18	0.58 (0.22–1.50)	0.26	0.46 (0.18–1.14)	0.09
Thailand	0.80 (0.42–1.50)	0.48	0.76 (0.32–1.82)	0.54	0.47 (0.21–1.04)	0.06
Vietnam	0.96 (0.50–1.83)	0.91	1.06 (0.45–2.49)	0.88	0.73 (0.37–1.44)	0.36
**Complication at delivery**	1.81 (1.36–2.39)	< 0.001	1.81 (1.37–2.40)	< 0.001	1.79 (1.35–2.38)	< 0.001
**Induced labour**	1.42 (0.98–2.05)	0.06	1.42 (0.98–2.07)	0.06	1.39 (0.96–2.01)	0.08
**Birth weight**		< 0.001		< 0.001		< 0.001
Low (< 2500 g)	0.59 (0.31–1.13)	0.11	0.58 (0.30–1.11)	0.10	0.60 (0.31–1.15)	0.12
Normal (2500-4000 g)	1		1		1	
Macrosomia (≥ 4000 g)	3.54 (2.05–6.12)	< 0.001	3.49 (2.02–6.05)	< 0.001	3.58 (2.07–6.19)	< 0.001
**Nulliparity**	3.23 (2.52–4.15)	< 0.001	3.22 (2.50–4.13)	< 0.001	3.22 (2.51–4.13)	< 0.001
**Attending ANC in another private facility**	1.28 (0.94–1.74)	0.11	1.31 (0.96–1.77)	0.08	1.26 (0.93–1.71)	0.14
**Urban residency**	0.78 (0.57–1.06)	0.12	0.75 (0.55–1.03)	0.08	0.79 (0.58–1.08)	0.15
**Maternal occupation**		0.01		0.01		0.01
Unemployed/housewife	1		1		1	
Employed formal sector	1.41 (1.02–1.94)	0.03	1.41 (1.02–1.94)	0.03	1.40 (1.02–1.93)	0.04
Informal sector	0.88 (0.65–1.19)	0.41	0.89 (0.66–1.20)	0.46	0.87 (0.65–1.78)	0.38
**Status of the maternity unit**						
Tertiary level of care	-	-	1.52 (0.72–3.20)	0.27	-	-
Private practice in the maternity unit	-	-	1.34 (0.69–2.60)	0.38	-	-
Teaching facility	-	-	0.74 (0.35–1.57)	0.44	-	-
Functioning US machine in delivery ward	-	-	0.79 (0.40–1.57)	0.51	-	-
Permanence of an anaesthetist	-	-	0.87 (0.48–1.54)	0.63	-	-
Any individual delivery room	-	-	1.14 (0.57–2.28)	0.71	-	-
**Workload and care capacity**						
Number of births per one midwife/nurse per day	-	-	-	-	0.79 (0.48–1.29)	0.35
Number of births per one obstetrician per day	-	-	-	-	0.97 (0.81–1.14)	0.69
Number of births per admission bed per day	-	-	-	-	0.77 (0.53–1.10)	0.15
Number of births per delivery bed per day	-	-	-	-	1.45 (1.09–1.93)	0.01
**Intraclass correlation**	-	-	0.06 (0.03–0.13)		0.05 (0.02–1.11)	

^a^Mixed-effects logistic regression models with random intercept

^b^Model 4: women’s characteristics; model 5: women’s characteristics and maternity unit status; model 6: women’s characteristics and indicators of workload and care capacity

**Fig. 5:**
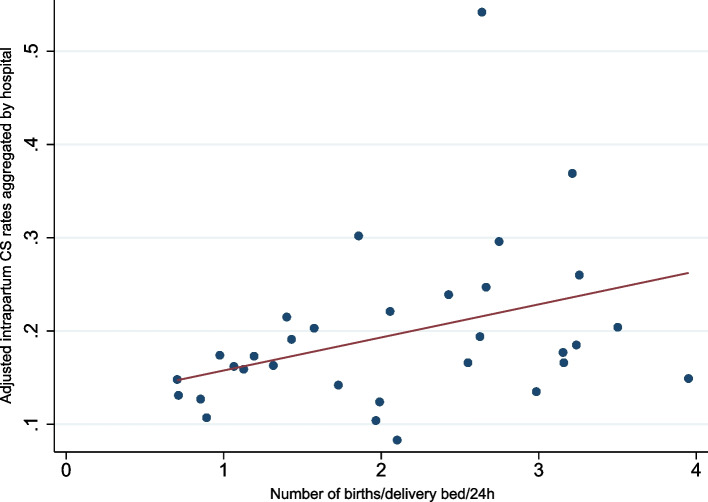
Relation between use of intrapartum CS and bed occupancy

Based on the elasticity estimation, intrapartum CS rate would increase of 6.3% points if the average number of births per delivery bed per day increased by 10% (elasticity = 0.63 [0.16; 1.09], *p* < 0.01). This result is illustrated in Fig. 5 where we plotted the aggregate intrapartum CS rate by hospital, calculated from the regression model (model 6) as a function of the number of births per delivery bed per day in the corresponding hospital. Interaction tests do not show any different impact of organizational factors on CS rates between countries, and between nulliparous and multiparous women.

## Discussion

This multi-site, multi-country study shows that CS use among low-risk women varied not only between countries, but also between hospitals within the same country. This variability is partly explained by individual and organizational factors. The availability of an anaesthetist fully dedicated to the maternity unit and the lack of individual delivery rooms in the hospital increased the use of pre-labour CS, while intra-partum CS increased when delivery bed occupancy increased as well.

Our analysis shows that CS rates were particularly high in those women who can be considered to be “lower risk” women from the perspective that they had a single, full-term pregnancy with cephalic presentation and no previous CS. These results are consistent with previous studies in LMICs that have shown that the population of low-risk women has high CS rates and is the main contributors to overall CS rates in these countries [3, 5, 34, 35].

In our study, there was one pre-labour CS for every four intra-partum CS. Fetal distress, cephalopelvic disproportion and failure to progress were the main indications for intra-partum CS. These finding are consistent with the results of previous studies in similar context [13, 36–38]. Most pre-labour CS were performed in Argentina and Thailand, countries where women’s preference for a pre-labour CS seems to be marked, revealed by women and accepted by obstetricians [39–41]. This is correlated with our findings which show that crude odds ratios of intrapartum CS are usually equal or smaller than those of pre-labour CS and that more than 30% of pre-labour CS were performed on maternal request in our sample. Accepting maternal request for CS without medical indication has been reported to be a way for obstetricians to avoid the potential litigation they fear and may be partly responsible for the high pre-labour CS rate in low-risk women [18, 22]. This practice might be reinforced by women’s preference for CS due to fear of pain during vaginal delivery but also to their perception of CS as a safe procedure [42–44].

In this study, women who gave birth in a participating hospital with a dedicated 24-hour anaesthetist in the maternity ward had an increased risk of pre-labour CS than in a hospital with an anaesthetist who shares his activity with other services. Similar results were found in Senegal and Mali [16]. Obviously, the full availability of an anaesthetist makes it easy to plan a CS, whether on medical indication or on maternal request. Although the presence of a dedicated anaesthetist is highly desirable to ensure that women who require a CS do not have difficulty accessing it, there is also a risk that hospitals perform excessive pre-labour CS routinely if no intervention is put in place to ensure quality of care and to reduce medically inappropriate surgery [11].

Our findings highlight the need to maintain a favourable environment in the maternity unit in order to improve quality of care and decrease unnecessary CS. Women who gave birth in a hospital with individual delivery rooms were less likely to give birth by pre-labour CS than women who gave birth in hospitals with only shared delivery rooms. Lack of privacy in shared delivery rooms is associated with a negative birth experience and women’s preference for pre-labour CS [43, 45]. The possibility to have an individual room reassures women of their privacy during delivery, usually allows a companion to be present by their side and thus might encourage them to attempt a vaginal birth. Additionally, we showed of positive relationship between intrapartum CS rates and delivery bed occupancy as a proxy of care capacity. This finding is not in line with that found in China [46], but is in accordance with two studies conducted in France [23, 32]. In this country, authors have shown that staffing levels and high number of births by delivery room also affect the use of caesarean section. Overcrowded maternity units is perceived by providers as a barrier to the quality of intrapartum care and may influence providers’ decision to perform a CS during labour when one could have waited longer [18]. Women who have had a poor experience during childbirth, relating to the conditions in shared rooms, can also request intrapartum CS for their next pregnancy [42].

This multi-site and multi-country design is a strength of this study that allows for the generalisation of results in similar contexts. Moreover, we carried out a comprehensive data collection on potential determinants of CS rates [31]. Our results show well known individual risk factors for pre-labour or intrapartum CS in LMICs: complications during pregnancy [16, 23, 47, 48], high birth weight [16, 23, 49, 50], maternal age [13, 16, 50–52], education [13, 50], employment [53].

Our study might have some limitations. Our sample of hospitals is not representative of all hospitals in the four participating countries. Our results only apply to hospitals with moderate to high caesarean section rates in in Burkina Faso, Argentina, Thailand and Vietnam. Moreover, organisational data such as bed occupancy levels or workload for healthcare providers were annual averages collected in 2020. These indicators may change over time and may not reflect the situation at the time of the post-partum survey. However, due to ongoing collaboration with researchers in countries who did not report significant changes, we believe that these variations, if they occurred, were minimal. In addition, our decision of not including institutional and organisational variables in the same statistical model (due to the collinearity), may have biased the elasticity measure due to a lack of adjustment for hospital characteristics.

Our results do not allow us to know whether women’s level of wealth is a determining factor in accessing individual rooms, but it is likely that the poorest women have less access to this type of room as compared with the richest. Anyway, wealth index was not significantly associated with pre-labour CS in our analysis which suggests that this “individual room” effect entails other components than the wealth index. Only two private hospitals were included (in Vietnam), compared to 30 public hospitals with or without private ward. Since several studies have shown that there is an association between private hospitals and CS use in LMICs [13, 14, 18, 25, 33, 50, 51], we may have underestimated CS rates among low-risk women in the participating countries and the effect of this private practice could not be identified. Nevertheless, our results show that women who attended ANC in another private facility have higher pre-labour CS rates than other women. Financial incentives, perceived better time management or obstetrician compliance with performing a CS on maternal request in the private sector may explain this association [13, 14, 18, 25, 33]. Finally, factors related to the healthcare providers, quality of care and compliance with medical guidelines were not taken into account in this study [18].

The results of this study have practical implications for policy makers, hospitals managers and healthcare providers to reduce unnecessary CSs, as evidenced by the high CS rates among low-risk women in the 32 participating hospitals. Firstly, in contexts where the availability of an anaesthetist fully dedicated to the maternity unit facilitates overuse of CS, efforts must be made to improve decision making of pre-labour CS [11]. To this end, non clinical interventions such as mandatory second-opinion, opinion leaders and peer-review of CS indications are recommended to reduce unnecessary CS [11, 54].

Secondly, to encourage women to attempt vaginal delivery when appropriate, hospitals should make effort to reassure women and offer them as much privacy as possible. Individual delivery room is optimal, but using curtains in shared delivery rooms could be an option [11]. More privacy will facilitate companionship during labour and delivery. This approach helps women reduce their anxiety, communicate with healthcare providers and deliver vaginally [55–57].

Regarding limited care capacity in referral hospital with high delivery rates, efforts should be made to adapt the number of delivery beds to the expected number of women in labour. Our results suggest that hospitals with between 1 and 2 births/bed/24 h is associated with lower intra-partum CS rates as compared with 3 births/bed/24 h. To this end, health policy should focus on the quality of care in level 1 or 2 hospitals and encourage women with low obstetrical risk to give birth in these facilities rather than in referral hospitals. Women could benefit from giving birth in smaller hospitals, possibly closer to their home, with the same quality of care as in large hospitals.

## Conclusion

Lack of privacy, easy access to surgery and limited intrapartum care capacity increase the use of CS in low risk women of participating hospitals. In this context it is crucial to improve the conditions and environment of the delivery room and the organisation of intrapartum care for a better birth experience. It is also important to implement non clinical interventions targeting health care professionals to improve decision-making regarding the mode of birth.

### Supplementary Information

**Additional file 1: Supplementary information Table S1. **Pre-defined list used to collect indication for CS (Quali-Dec post-partum survey).

**Additional file 2: Supplementary Figure S1.** Flow-chart representing the recruitment of participants into the baseline Quali-Dec survey.

## Data Availability

The datasets generated and/or analysed during the current study will be available on Zenodo (https://www.zenodo.org/) under the community “QUALI-DEC - Appropriate use of Caesarean section through QUALIty DECision-making by women and providers (847567)”. The database anonymisation process is underway and run by the principal data manager of the project. All datasets relating to the Quali-Dec baseline survey will be available by the end of the project (end 2024). Zenodo is a general-purpose open-access repository developed under the European OpenAIRE program and operated by the European Organization for Nuclear Research (CERN). Zenodo will allow the deposition of datasets, reports, and any other digital artifacts related to research. For each repository, a persistent DOI will be created to easily cite the stored items. The metadata of each record will be indexed and searchable directly in Zenodo’s search engine immediately after publishing.

## Abbreviations

CS: Caesarean section
LMICs: Low- and middle-income countries
ANC: Antenatal care
DAT: Decision analysis tool
WHO: World Health Organization
REDCap: Research electronic data capture
OR: Odds ratio
SD: Standard deviation
CI: Confidence interval
ORa: Adjusted odds ratio
