# Supplementary material for: How does hospital organisation influence the use of caesarean sections in low- and middle-income countries? A cross-sectional survey in Argentina, Burkina Faso, Thailand and Vietnam for the QUALI-DEC project
Source: BMC Pregnancy Childbirth. 2024 Jan 17;24:67. doi: 10.1186/s12884-024-06257-w (PMC10792793; doi:10.1186/s12884-024-06257-w)
Supplement: Supplementary file 1 — Additional file 1: Supplementary information Table S1. Pre-defined list used to collect indication for CS (Quali-Dec post-partum survey). [file 12884_2024_6257_MOESM1_ESM.docx]

**Supplementary information Table S1:** Pre-defined list used to collect indication for CS (Quali-Dec post-partum survey)

| **If caesarean section, please select the indications below :** | **Yes/No** |
| --- | --- |
| Suspected fetal growth impairment |  |
| Fetal distress |  |
| Pre-eclampsia |  |
| Eclampsia |  |
| Gestational age 41 completed weeks or more |  |
| Third trimester vaginal bleeding |  |
| Cephalopelvic disproportion |  |
| Dystocia/failure to progress |  |
| Failed vacuum extraction or forceps |  |
| Multiple pregnancy |  |
| Medically-assisted procreation |  |
| Suspected/imminent uterine rupture |  |
| Postmortem caesarean section |  |
| Breech or other malpresentation |  |
| Previous caesarean section |  |
| Failed induction |  |
| Tubal ligation/sterilization |  |
| Maternal request |  |
| HIV |  |
| Genital Herpes/extensive condyloma |  |
| Previously repaired vesiculo-vaginal or recto-vaginal fistula |  |
| Previous uterine surgery |  |
| Any other indication |  |
