# Supplementary material for: How does hospital organisation influence the use of caesarean sections in low- and middle-income countries? A cross-sectional survey in Argentina, Burkina Faso, Thailand and Vietnam for the QUALI-DEC project
Source: BMC Pregnancy Childbirth. 2024 Jan 17;24:67. doi: 10.1186/s12884-024-06257-w (PMC10792793; doi:10.1186/s12884-024-06257-w)
Supplement: Supplementary file 2 — Additional file 2: Supplementary Figure S1. Flow-chart representing the recruitment of participants into the baseline Quali-Dec survey. [file 12884_2024_6257_MOESM2_ESM.docx]

Supplementary Figure S1: Flow-chart representing the recruitment of participants into the baseline Quali-Dec survey

**Women included for analysis
N = 2,092**- Burkina Faso, n = 407
- Argentina, n = 440
- Thailand, n = 572
- Vietnam, n = 673

Women excluded due to specific exclusion criteria
N = 1,035
Non-cephalic presentation (n=130), multiple pregnancy (n=69), history of CS (n=659) and pre-term delivery (n=254); Emergency pre-labour caesarean section (n=57)

**Consent to participate**

**Women interviewed 
N = 3,127**-   Burkina Faso, n = 686
-   Argentina, n = 626
-   Thailand, n = 859
-   Vietnam, n = 956

Women excluded because of incomplete forms
N = 8

**Women who consented to participate  
N = 3,135**-   Burkina Faso, n = 687
-   Argentina, n = 626
-   Thailand, n = 859
-   Vietnam, n = 963

Women who didn't consent 
N = 201

**Women who were approached for interview 
N = 3,336**-   Burkina Faso, n = 689
-   Argentina, n = 686
-   Thailand, n = 922
-   Vietnam, n = 1,039

Women excluded (health
problems/giving birth to a malformed, stillborn or deceased child)
N = 685

Women not approached for the interview
N = 16

**Approach to women**

**Women found eligible to participate
N = 3,352**-   Burkina Faso, n = 695
-   Argentina, n = 688
-   Thailand, n = 926
-   Vietnam, n = 1,043

**Assessment of eligibility (screening form)**

**Selected women in the 32 hospitals
N = 3,534**-   Burkina Faso, n = 819 
-   Argentina, n = 715
-   Thailand, n = 949
-   Vietnam, n = 1,051

Women randomly excluded
N = 2,306

**Randomization process**

**Women who gave birth during the data collection period in the 32 hospitals
N = 5,840**-   Burkina Faso, n = 1,320
-   Argentina, n = 715
-   Thailand, n = 1,035
-   Vietnam, n = 2,770
